# Cerebral glucose metabolic correlates of cognitive and behavioural impairments in amyotrophic lateral sclerosis

**DOI:** 10.1007/s00415-024-12388-z

**Published:** 2024-06-11

**Authors:** Annaliis Lehto, Julia Schumacher, Elisabeth Kasper, Stefan Teipel, Andreas Hermann, Jens Kurth, Bernd Joachim Krause, Johannes Prudlo

**Affiliations:** 1 Department of Neurology, Translational Neurodegeneration Section “Albrecht Kossel”, Rostock University Medical Center, Rostock, Germany; 2 Deutsches Zentrum für Neurodegenerative Erkrankungen (DZNE), Rostock-Greifswald, Rostock, Germany; 3 Department of Neurology, Rostock University Medical Center, Rostock, Germany; 4 Department of Psychosomatic Medicine, Rostock University Medical Center, Rostock, Germany; 5 Department of Nuclear Medicine, Rostock University Medical Center, Rostock, Germany

**Keywords:** Amyotrophic lateral sclerosis, Cerebral glucose metabolism, FDG-PET, Cognition, Grey matter volume

## Abstract

**Objective:**

Half of ALS patients are cognitively and/or behaviourally impaired. As cognition/behaviour and cerebral glucose metabolism can be correlated by means of ^18^F-Fluorodeoxyglucose positron emission tomography (FDG-PET), we aimed to utilise FDG-PET, first, to replicate group-level differences in glucose metabolism between non-demented ALS patients separated into non-impaired (ALSni), cognitively impaired (ALSci), behaviourally impaired (ALSbi), and cognitively and behaviourally impaired (ALScbi) groups; second, to investigate glucose metabolism and performance in various cognitive domains; and third, to examine the impact of partial volume effects correction (PVEC) of the FDG-PET data on the results.

**Methods:**

We analysed neuropsychological, clinical, and imaging data from 67 ALS patients (30 ALSni, 21 ALSci, 5 ALSbi, and 11 ALScbi). Cognition was assessed with the Edinburgh Cognitive and Behavioural ALS Screen, and two social cognition tests. FDG-PET and structural MRI scans were acquired for each patient. Voxel-based statistical analyses were undertaken on grey matter volume (GMV) and non-corrected vs. PVE-corrected FDG-PET scans.

**Results:**

ALSci and ALScbi had lower cognitive scores than ALSni. In contrast to both ALSni and ALSci, ALScbi showed widespread hypometabolism in the superior- and middle-frontal gyri in addition to the right temporal pole. Correlations were observed between the GMV, the FDG-PET signal, and various cognitive scores. The FDG-PET results were largely unaffected by PVEC.

**Interpretation:**

Our study identified widespread differences in hypometabolism in the ALScbi-ni but not in the ALSci-ni group comparison, raising the possibility that cerebral metabolism may be more closely related to the presence of behavioural changes than to mild cognitive deficits.

**Supplementary Information:**

The online version contains supplementary material available at 10.1007/s00415-024-12388-z.

## Introduction

Amyotrophic lateral sclerosis (ALS) is a progressive neurodegenerative motor neuron disorder. Besides rapidly advancing motor impairment, up to 50% of people living with ALS are cognitively impaired, up to 70% show behavioural changes, and up to 15% meet the criteria for a frontotemporal dementia [1]. The cognitive domains characteristically affected in ALS include verbal fluency, executive functioning, social cognition, and language [2, 3]. Behavioural changes include apathy, irritability, and increased self-centeredness [1, 4, 5].

Positron emission tomography (PET) imaging with the radiotracer 2-[^18^F]Fluorodeoxyglucose (FDG) provides an estimation of glucose uptake by neurons and astrocytes [6, 7], enabling the investigation of differences in regional metabolism. The typical pattern of glucose hypo- and hypermetabolism in ALS has been well documented, with the most consistently reported hypometabolic regions being the motor cortex and prefrontal cortex [8–10]. In terms of hypermetabolism, previous studies have reported alterations in the cerebellum, occipital cortex, midbrain, and medial temporal cortex [8–12].

Associations have been reported between cognitive status and metabolic changes in ALS patients: more extensive and severe frontal hypometabolism has been correlated with an increased severity of cognitive and behavioural impairments [13]. Besides group differences between ALS patients with or without cognitive impairments, correlations have been described between metabolism in specific brain regions and performance in cognitive tasks. An impaired cognitive theory of mind has been linked to increased metabolism in the left fusiform gyrus [10], and decreased metabolism in the prefrontal cortex [14], the supplementary motor area [14], and the left inferior frontal gyrus [15]. Weaker episodic memory performance has been associated with increased metabolism in the hippocampus and left parahippocampal gyrus [10]. Concerning behavioural impairment, the increasing severity of apathy has been negatively associated with metabolism in the prefrontal cortex, premotor cortex, anterior cingulate gyrus, and insula as well as positively associated with metabolism in the cerebellum and pons [16]. However, evidence is still scarce on associations between glucose metabolism and specific cognitive domains relevant in ALS such as executive functioning, verbal fluency, and social cognition.

An important consideration for PET analyses are partial volume effects (PVEs). The radiotracer signal measured in a given brain region reflects a combination of the true regional signal and the signal from neighbouring tissues or CSF, leading to blurring [17]. When the signal in grey matter is higher than in white matter or CSF, as is the case with FDG-PET, PVEs lead to an underestimation of the true grey matter signal. Besides scanner resolution, the extent of PVEs is further dependent on the size of the measured region and the presence of atrophy [17]. The majority of FDG-PET studies did not include a PVE correction (PVEC) [8, 9, 11, 13, 16] based on the assumption that metabolic measurements in ALS are relatively unaffected by PVEs, but this assumption has not yet widely been tested.

The aims of this study were thus (1) to replicate findings on differences in glucose metabolism and grey matter volume between ALS patients with and without cognitive or behavioural impairment, (2) to investigate associations of glucose metabolism and grey matter volume with performance in ALS-related cognitive domains, and (3) to examine the impact of PVEC of the FDG-PET data on the group differences and domain-specific associations.

## Materials and methods

### Participants

67 patients were recruited by the Department of Neurology of the Rostock University Medical Centre. They were diagnosed according to the modified El Escorial criteria [18] and characterised using the revised ALS Functional Rating Scale (ALSFRS-R) [19]. The exclusion criteria included a history of brain injury, epilepsy, psychiatric illness, or a diagnosis of dementia [20].

### Neuropsychological assessment

The neuropsychological assessment included the German versions of the Edinburgh cognitive and behavioural ALS screen (ECAS) [21], Faux Pas test [22], the Facial Recognition Task [23], and the informant version of the Frontal System Behaviour Scale (FrSBe) [24]. ECAS assesses five cognitive domains with multiple subtests [25], the Faux-pas test is an advanced theory of mind task, and the Facial Recognition Task measures the ability to identify the six basic emotions based on facial expressions. The FrSBe assesses behavioural changes in apathy, disinhibition, and executive dysfunction.

Cognitive and behavioural impairments were classified according to the revised Strong criteria [26] as follows: patients without cognitive and/or behavioural impairment (ALSni), patients with cognitive impairment (ALSci), patients with behavioural impairment (ALSbi) and patients with cognitive and behavioural impairment (ALScbi). Cognitive impairment was defined as a deficient score in the verbal fluency, executive functions, or language domain based on age- and education-adjusted ECAS-norms from a Swiss and German sample [21]. Norms from a French sample were used for Faux Pas test and Facial Recognition Test [27]. The behavioural classification was based on either or both the age- and education-normed informant version of FrSBe [24], and the clinical observation of an experienced neuropsychologist.

### Neuroimaging data acquisition

Structural MRI scans were taken on two scanners and FDG-PET scans were acquired on the same scanner at the Rostock University Medical Centre (Rostock, Germany). High-resolution T1-weighted anatomical images were obtained on a 3 T Siemens Skyra Fit scanner (2018–2022) or on a Siemens Vida scanner (from 2022 onwards) using the magnetization-prepared rapid gradient echo (MPRAGE) sequence with the following parameters: 192 sagittal slices, field of view = 256 × 256 mm^2^, image matrix = 256 × 256, isotropic voxel size = 1 mm^3^, echo time = 4.37 ms, repetition time = 2500 ms, and flip angle = 7°.

The patients underwent dynamic PET imaging (4 × 5 min) of the brain using a Gemini TF 16 scanner (Philips Healthcare) at 30 min after the injection of 199 ± 18 MBq FDG. Prior to PET imaging, an auxiliary CT scan (120 kVp, 30 mAs) was performed. To minimise the influence of motion artefacts, the four dynamic PET frames were examined for possible head movements of the patient. Conspicuous frames were excluded, and finally, a static PET data set, representing at least 10 min acquisition time, was reconstructed using the manufacturer's proprietary BLOB-OS reconstruction algorithm (3 iterations, 31 subsets) corrected for randoms, scatter, decay, and attenuation using information from the auxiliary CT.

### Neuroimaging preprocessing

All imaging data were preprocessed in SPM12 (https://www.fil.ion.ucl.ac.uk/spm/) using Matlab.

*MRI:* The MRI scans were skull-stripped, segmented, and spatially normalised to the MNI152 non-linear asymmetric 2009 template using Dartel registration in the CAT12 toolbox [28]. The grey matter (GM), white matter (WM), and CSF segments in addition to the forward and inverse deformation fields were saved. The scans were smoothed with a 8 mm kernel and submitted to statistical analysis. In addition, an explicit GM mask was created by keeping GM voxels that overlap in at least 50% of the spatially normalised scans and applied in all statistical analyses.

*FDG-PET:* The FDG-PET scan was coregistered to the participant’s MRI scan and preprocessed both with and without PVEC according to the Müller-Gärtner [29] method using custom Matlab scripts to enable a comparison of results. GM, WM, and CSF segments from the MRI preprocessing were smoothed with a 4.8 mm kernel, accounting for the spatial resolution of the used PET-system [30], and used in the PVEC procedure. A GM threshold of 0.5 was incorporated, and trilinear interpolation was used. For the PVEC procedure, WM and CSF signal was estimated for each individual patient by thresholding their WM and CSF segments at 0.99 and averaging the signal in the resulting mask. The output of PVEC was a map of corrected FDG signal restricted to GM. Negative voxels were masked-out, and to compare results with and without PVEC the negative and non-GM voxels were also masked-out in the non-corrected FDG-PET (NC-FDG) scans. Intensity normalization on both PVEC-FDG and NC-FDG scans was carried out by dividing the signal of each voxel by the average FDG signal within GM [13, 15, 16]. This intensity normalisation approach impacts the interpretation of hypermetabolism: when global metabolism is reduced throughout widespread hypometabolic regions, then clusters of hypermetabolism may instead indicate a relative preservation of metabolism. Subsequently, all scans were spatially normalised to MNI space by applying forward deformation fields from MRI preprocessing step with trilinear interpolation. All scans were smoothed with a 10 mm kernel and submitted to statistical analysis.

### Statistical analysis

The whole sample was characterised by various demographic and clinical data and equivalence of the groups was compared using Chi-squared tests for categorical variables and Analysis of Variance (ANOVA) for continuous variables in the Statistical Product and Service Solutions (SPSS, version 28.0). Kruskal–Wallis tests were used to compare the groups on individual cognitive domains. For cognitive domains where the groups differed, individual subtest scores contributing to the domain score were also compared. Mann–Whitney U tests were used for all post hoc comparisons.

Group differences in PVEC-FDG data, NC-FDG data, and GM volume were tested in a voxel-based manner with one-way ANOVA in SPM12 using Matlab. Age and sex were included as covariates in all analyses, and the total intracranial volume (TIV) was included in GM volume analysis. All possible t-contrasts were tested. Further voxel-based regression analyses on the PVEC-FDG signal, NC-FDG signal, and GM volume were specified in SPM12 for single cognitive domains and subtest scores that differed in the preceding neuropsychology analyses. Age, sex, and years of formal education were included as covariates, and the total intracranial volume (TIV) was included in GM volume analysis.

In addition, corresponding analyses were carried out using partial least squares (PLS) regression with the PLS application in Matlab [31]. This method makes use of covariance between voxels and yields latent variables (LVs) that each identify a pattern of brain regions that covary with some external measure [32]. The significance of each LV was determined with 100 permutation tests at a threshold of *p* < 0.05. Each voxel has a salience on each LV. To determine the reliability of these voxel saliences, we used the bootstrap estimation with 100 iterations and a threshold of *p* < 0.05 for the resulting bootstrap ratios. We examined all group contrasts in addition to the associations with performance on cognitive domains that differed in the preceding neuropsychology analyses using the PVEC-FDG and NC-FDG data.

## Results

### Demographic and clinical characteristics

The clinical and demographic information for our sample can be found in Table 1. Based on the small group size of ALSbi (*n* = 5), they were excluded from the group analysis. The equivalence of the groups on different variables was tested using Chi-squared tests for categorical and ANOVAs for quantitative variables.

**Table 1:** Clinical and demographic characteristics

	ALSni	ALSci	ALScbi	Total	*p*-value
Group size: count (%)	30 (48.4%)	21 (33.9%)	11 (17.7%)	62 (100%)	**0.013**
Age: M (SD)	62.1 (12.9)	69.9 (7.9)	67.3 (9.8)	65.7 (11.3)	**0.045**
Sex: female/male	9/21	10/11	2/9	21/41	0.204
Education years: M (SD)	14.6 (2.9)	13.0 (2.4)	13.4 (2.8)	13.8 (2.8)	0.106
Months since onset: median (IQR)	14 (9.0–24.8)	9 (6.0–19.0)	13 (4.5–27.5)	12.5 (7.0–24.0)	0.337
Site of onset: count (%)					0.482
Spinal onset	24 (80.0%)	14 (66.7%)	9 (81.8%)	47 (75.8%)	
Bulbar onset	6 (20.0%)	7 (33.3%)	2 (18.2%)	15 (24.2%)	
Phenotype: count					0.907
Classical ALS	23	15	9	47	
PLS	1	2	0	3	
UMND	2	2	1	5	
PMA	2	1	1	4	
LMND	2	0	0	2	
Not classifiable	0	1	0	1	
ALSFRS-R: median (IQR)	39.0 (36.3–41.8)	39.0 (36.0–40.0)	35.0 (33.0–41.0)	39.0 (35.0–41.0)	0.460
Genetic status (count)					
C9ofr72	2	1	1	4	
TARDBP	0	1	0	1	
SOD1	0	0	1	1	
No mutation	15	9	5	29	
Untested	13	10	4	27	

*ALSni* not impaired; *ALSci* cognitively impaired; *ALScbi* cognitively and behaviourally impaired; *M* mean; *SD* standard deviation; *IQR* interquartile range; *PLS* primary lateral sclerosis; *UMND* upper motor neuron-dominant ALS; *PMA* progressive muscular atrophy; *LMND* lower motor neuron-dominant ALS

### Neuropsychological data

The neuropsychological data are summarised in Table 2. The three groups were compared using Kruskal–Wallis and Mann–Whitney tests. For single cognitive domains where the groups differed, subtest scores were also compared. The ALSni and ALSci patients differed on the cognitive domains of language, executive functions, verbal fluency, and memory, whereas differences between ALSni and ALScbi patients were observable on executive functions, verbal fluency, and memory. No differences in cognitive scores were observed between ALSci and ALScbi patients.

**Table 2:** Neuropsychological results

		ALSni vs ALSci		ALSni vs ALScbi		ALSci vs ALScbi	
Dependent variable	Test statistic (*p*-value)^1^	Test statistic (*p*-value)^2^	Effect size^3^	Test statistic (*p*-value)^2^	Effect size^3^	Test statistic (*p*-value)^2^	Effect size^3^
ECAS: Language	**8.2 (0.016)**	**180 (0.008)**	0.373	102 (0.053)	0.302	107 (0.732)	0.060
Naming	3.4 (0.182)						
Comprehension	3.7 (0.156)						
Spelling	**9.2 (0.010)**	**209.5 (0.026)**	0.311	**76.5 (0.004)**	0.444	98.5 (0.490)	0.122
ECAS: Executive functions	**15.8 (0.001)**	**135 (0.001)**	0.483	**64 (0.003)**	0.466	106 (0.704)	0.054
Digit span	**8.0 (0.019)**	**213.5 (0.049)**	0.275	**82 (0.013)**	0.388	87.5 (0.255)	0.201
Alternation	**8.2 (0.016)**	**199 (0.015)**	0.342	**92 (0.017)**	0.372	114.5 (0.967)	0.007
Inhibition	**7.4 (0.024)**	**193 (0.017)**	0.335	**99 (0.045)**	0.313	102 (0.583)	0.097
Social cognition	**14.0 (0.001)**	**196 (0.002)**	0.444	**97 (0.002)**	0.477	110 (0.810)	0.043
ECAS: Verbal fluency	**35.1 (0.001)**	**34 (0.001)**	**0.878**	**31.5 (0.001)**	**0.621**	97 (0.450)	0.133
S-words	**27.7 (0.001)**	**541.5 (0.001)**	**0.607**	**311 (0.001)**	**0.671**	111.5 (0.874)	0.028
G-words	**30.0 (0.001)**	**578 (0.001)**	**0.705**	**286.5 (0.001)**	**0.558**	86.5 (0.248)	0.204
ECAS: Memory	**10.6 (0.005)**	**163.5 (0.004)**	0.407	**88 (0.023)**	0.355	97 (0.460)	0.130
Immediate recall	**13.6 (0.001)**	**156 (0.002)**	0.430	**69 (0.004)**	0.446	92 (0.344)	0.167
Delayed recall	**11.4 (0.003)**	**161 (0.003)**	0.418	**82.5 (0.014)**	0.384	100 (0.533)	0.110
Recognition	5.2 (0.075)						
ECAS: Visuo-spatial	0.6 (0.732)						
Faux Pas test	**8.2 (0.017)**	**55.5 (0.024)**	0.405	**36 (0.025)**	0.424	29.5 (0.230)	0.275
Facial recognition task	3.6 (0.165)						
FrSBe: total	**6.9 (0.031)**	54.5 (0.616)	0.112	**63 (0.010)**	**0.598**	**39.5 (0.045)**	**0.548**
Apathy	**9.1 (0.010)**	50 (0.877)	0.035	**67 (0.004)**	**0.685**	**42.5 (0.017)**	**0.639**
Disinhibition	2.2 (0.340)						
Executive dysfunction	4.9 (0.87)						

*ALSni* not impaired; *ALSci* cognitively impaired; *ALScbi* cognitively and behaviourally impaired. ^1^*H* statistic of the Kruskal–Wallis Test with 3 groups (asymptotic 2-tailed significance), ^2^*U* statistic of the Mann–Whitney Test (asymptotic 2-tailed significance), ^3^Pearson *r*

*P*-values < 0.05 and effect sizes > 0.5 are bold

### Group analyses

Groups were compared on GM volume, PVEC-FDG data, and NC-FDG data in a voxel-based manner. The height threshold was set at *p* < 0.001, *p* < 0.05 FWE-corrected at cluster level. If no clusters were detected, an exploratory analysis at a lenient threshold of *p* < 0.001 and a minimum cluster size of 100 voxels was undertaken (supplementary Fig. 1). The FWE-corrected results are reported below and both FWE-corrected and exploratory findings can be found in Tables 3 and 4. In addition, PLS analyses of the contrasts were carried out.

**Table 3:** Clusters of hypo- and hypermetabolism in ALScbi compared to ALSni patients

Data	Label	Side	*K*	*x*	*y*	*z*	*P* (uncorr. at cluster level)	*P* FWE (corr. at cluster level)
Hypometabolism								
PVEC-FDG	Middle frontal gyrus	*R*	5212	38	33	46	**0.000**	**0.000**
PVEC-FDG	Superior frontal gyrus	*L*	1214	– 10	52	26	**0.006**	**0.034**
PVEC-FDG	Temporal pole	*R*	1143	44	20	-33	**0.007**	**0.041**
PVEC-FDG	Supramarginal gyrus	*R*	504	58	– 36	42	0.058	0.283
PVEC-FDG	Triangular inferior frontal gyrus	*R*	264	44	38	3	0.157	0.594
NC-FDG	Superior frontal gyrus	*R*	4943	9	68	18	**0.000**	**0.000**
NC-FDG	Temporal pole	*R*	1443	44	20	-34	**0.003**	**0.016**
NC-FDG	Superior frontal gyrus	*L*	1109	– 22	62	0	**0.007**	**0.042**
NC-FDG	Superior frontal gyrus	*L*	447	– 10	52	26	0.068	0.329
NC-FDG	Triangular inferior frontal gyrus	*R*	424	52	38	9	0.074	0.355
NC-FDG	Supramarginal gyrus	*R*	367	58	– 38	42	0.094	0.427
Hypermetabolism								
PVEC-FDG	Superior occipital gyrus	*R*	2253	20	– 87	20	**0.000**	**0.003**
PVEC-FDG	Cerebellar lobule VI	*R*	746	26	– 72	– 18	**0.025**	0.133
PVEC-FDG	Cerebellar lobule VI	*L*	623	– 32	– 69	– 20	0.038	0.194
PVEC-FDG	Middle occipital gyrus	*L*	321	– 34	– 94	– 6	0.122	0.502
PVEC-FDG	Superior occipital gyrus	*L*	226	– 16	– 88	18	0.189	0.661
NC-FDG	Cerebellar lobule VI	*R*	586	26	– 72	– 18	**0.040**	0.209
NC-FDG	Fusiform gyrus	*L*	496	– 36	– 70	– 16	0.056	0.281
NC-FDG	Superior occipital gyrus	*R*	412	16	– 92	20	0.078	0.369
NC-FDG	Cuneus	*L*	406	– 9	– 92	14	0.080	0.377
NC-FDG	Superior occipital gyrus	*L*	214	– 16	– 87	18	0.192	0.679
NC-FDG	Middle occipital gyrus	*L*	213	– 36	– 94	– 8	0.193	0.681

*K* cluster size in voxels; *FWE* family wise error; *PVEC-FDG* PVE-corrected FDG-PET data; *NC-FDG* non-corrected FDG-PET data; *R* right; *L* left. Only clusters with more than 200 voxels are presented

**Table 4:** Clusters of hypo- and hypermetabolism in ALScbi compared to ALSci patients

Data	Label	Side	*K*	*x*	*y*	*z*	*P* (uncorr. at cluster level)	P FWE (corr. at cluster level)
Hypometabolism								
PVEC-FDG	Temporal pole	R	500	42	21	– 33	0.059	0.287
PVEC-FDG	Superior frontal gyrus	R	469	33	62	20	0.066	0.317
NC-FDG	Superior frontal gyrus	R	509	33	60	21	0.053	0.269
NC-FDG	Temporal pole	R	502	40	22	– 34	0.054	0.275
Hypermetabolism								
PVEC-FDG	Cuneus	L	1088	– 8	– 87	15	**0.009**	**0.048**
NC-FDG	Cuneus	L	1460	– 8	– 87	15	**0.003**	**0.016**

*K* cluster size in voxels; *FWE* family wise error; *PVEC-FDG* PVE-corrected FDG-PET data; *NC-FDG* non-corrected FDG-PET data; *R* right; *L* left. Only clusters with more than 200 voxels are presented

#### ALSni vs ALSci

No clusters were detected in GM volume, PVEC-FDG, nor the NC-FDG analysis. No differences could be detected in PLS analyses with both PVEC-FDG data (LV = 93.88, *p* = 0.574) and NC-FDG data (LV = 92.18, *p* = 0.614).

#### ALSni vs ALScbi

**Fig. 1:**
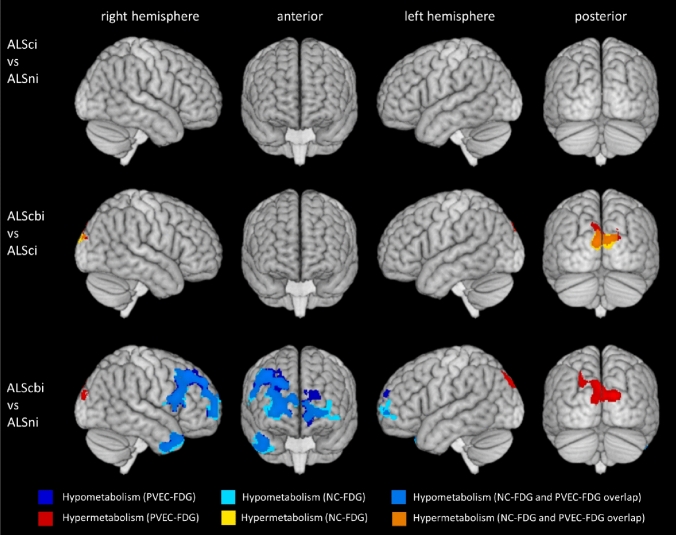
Clusters of relative hypo- and hypermetabolism per group comparison. The FDG-PET signal is relative to the average FDG-tracer uptake in GM. Height threshold is *p* < 0.001 and all data are *p* < 0.05 FWE-corrected at cluster level. *ALSni* not impaired; ALSci, cognitively impaired; *ALScbi* cognitively and behaviourally impaired; *PVEC-FDG* PVE-corrected FDG-PET data; *NC-FDG* non-corrected FDG-PET data

For PVEC-FDG and NC-FDG data, ALScbi patients showed hypometabolic clusters in the left superior frontal gyrus and right temporal pole. In PVEC-FDG analyses, the ALScbi group further showed hypometabolism in the right middle-frontal gyrus, and hypermetabolism in right superior occipital gyrus. The clusters are listed in Table 3 and portrayed on Fig. 1. The PLS analyses corroborated these results by identifying a similar metabolic pattern in both PVEC-FDG data (LV = 135.69, *p* < 0.001) and NC-FDG data (LV = 135.72, *p* < 0.001).

#### ALSci vs ALScbi

In PVEC-FDG and NC-FDG analyses, the ALScbi group showed a cluster of relative hypermetabolism in the left cuneus. The clusters are listed in Table 4 and portrayed on Fig. 1. In the PLS analyses, the difference was on the threshold of significance based on both the NC-FDG data (LV = 104.61, *p* = 0.050) and the PVEC-FDG data (LV = 99.71, *p* = 0.059).

### Associations with single cognitive domains and subtests

**Fig. 2:**
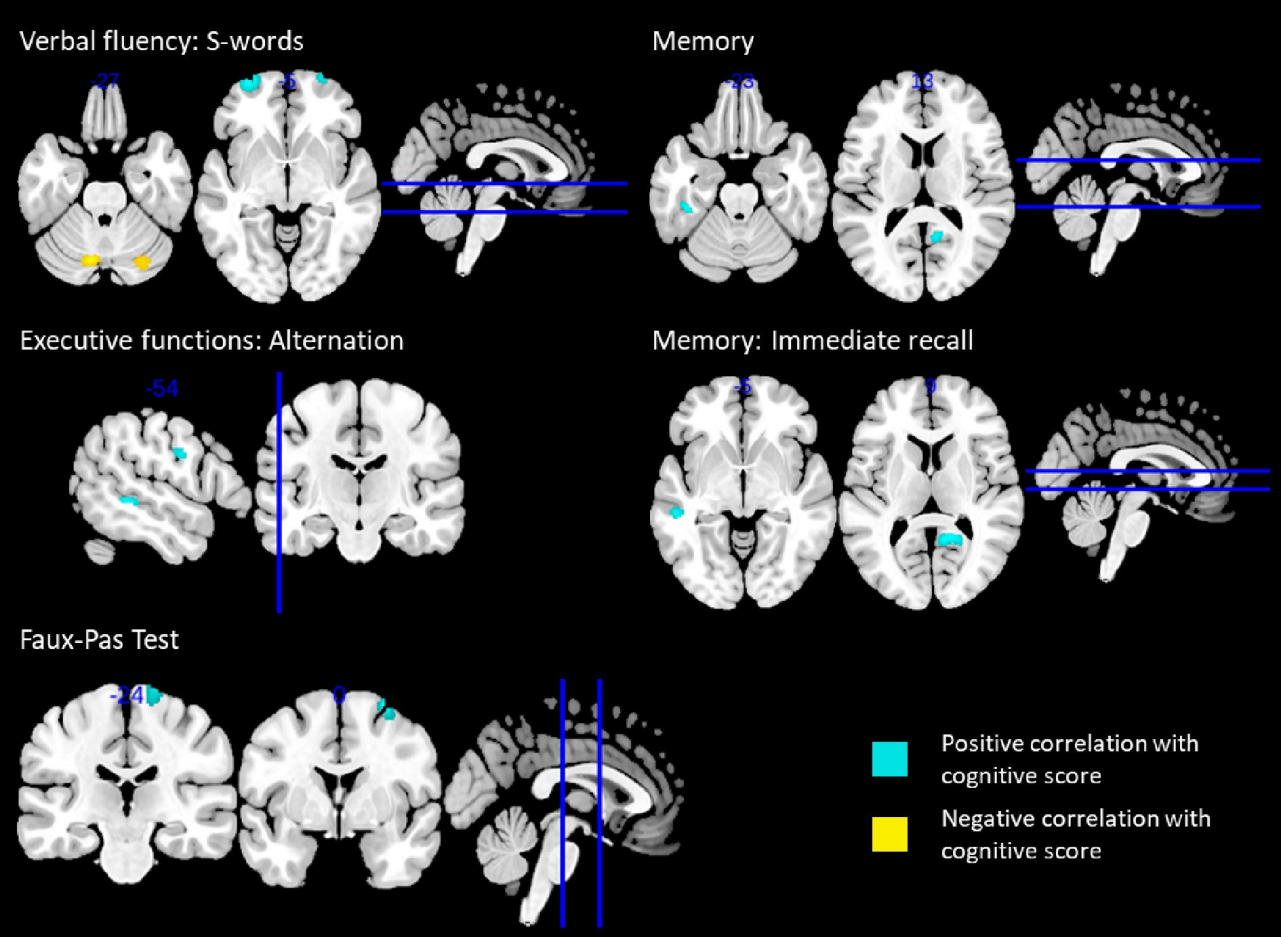
Correlations of glucose metabolism and cognitive scores. The FDG-PET signal is relative to the average FDG-tracer uptake in GM. Height threshold is *p* < 0.001 and minimum cluster size is 100 voxels

In the PVEC-FDG and NC-FDG analyses, no clusters were detected with FWE-correction at cluster level. Further exploratory analyses at a height threshold of *p* < 0.001 and a minimum cluster size of 100 voxels identified the following associations between glucose metabolism and single cognitive domains and their subtests (Fig. 2). All correlations were positive unless otherwise specified. The domain score of executive functioning correlated with metabolism in the left middle-frontal gyrus (NC-FDG), whereas its subtest inhibition correlated with left lingual gyrus metabolism (NC-FDG), and the subtest alternation with metabolism in the left lobule VIII of the cerebellum (NC-FDG), the left precentral gyrus (PVEC-FDG), and the left middle temporal gyrus (PVEC-FDG). The verbal fluency subtest of S-words correlated with metabolism in the bilateral superior frontal (PVEC-FDG and NC-FDG), left precentral (PVEC-FDG), and right inferior orbital gyrus (NC-FDG). Furthermore, negative associations were observed between the S-words score and metabolism in the bilateral crus I of the cerebellum. Memory performance was associated with metabolism in the right precuneus (PVEC-FDG), and the left fusiform gyrus (PVEC-FDG and NC-FDG) and the subtest of immediate recall was associated with the right precuneus (PVEC-FDG and NC-FDG) and the left middle temporal gyrus metabolism (PVEC-FDG and NC-FDG). Faux Pas test scores correlated with metabolism in the right middle-frontal gyrus (PVEC-FDG and NC-FDG) and right precentral gyrus (PVEC-FDG). None of the PLS analyses with PVEC-FDG or NC-FDG data neared significance (data not shown). Clusters of GM volume are outlined in supplementary Table 1.

## Discussion

The aim of this study was to investigate the associations between cerebral glucose metabolism and cognition in ALS using group comparisons and regression analyses. In accordance with previous work in the field [13], we found that concurrent cognitive and behavioural impairment were associated with widespread changes in glucose metabolism. Relative to the average cortical FDG-PET signal, hypometabolism in the superior frontal gyri, middle-frontal gyri, and temporal pole in addition to relative hypermetabolism in the superior occipital gyrus was detected in ALScbi when compared to ALSni. In exploratory analyses without FWE-correction, the comparison of ALScbi to ALSci identified a similar pattern of metabolism changes where the location of the clusters persisted, but their extent was conspicuously limited. This pattern did not emerge for the group comparison of ALSci to ALSni, even in the exploratory analysis without FWE-correction. The result of the partial least squares (PLS) analysis further corroborated these group comparison findings. The FDG-PET signal was intensity normalised using the average FDG signal in GM, which impacts the interpretation of hypermetabolic clusters. In the case of widespread hypometabolic areas leading to a reduction in global metabolism, the co-occurring clusters of hypermetabolism should be interpreted as a relative preservation of metabolism.

In contrast to both our expectations and a previous publication [13], no clusters of hypometabolism survived the FWE-correction in ALSci compared to ALSni patients in the current sample. One possible explanation for this lack of stark differences may be the heterogeneous composition of the ALSci as defined by Strong [26], since this category includes patients with mild deficits in either verbal fluency, executive functioning, or language, and those impairments may have different neural substrates. A further consideration is the use of the ECAS [25] instead of a comprehensive neuropsychological test battery (owing to both its feasibility and its lower burden for patients). The prevalence of deficits in ALS-specific cognitive functions (fluency, executive, and language) in our sample was comparable to the original publication, which was based on and validated in UK samples [25]. However, recent research has highlighted how crucial the choice of population norms and statistical methods are in determining the prevalence of cognitive impairment based on the ECAS [33]. The results of the group comparisons in our sample are, therefore, inherently dependent on the available age- and education-adjusted German ECAS-norms [21]. This raises questions of eligibility to directly compare our results with other cohorts and has implications for the generalisation of our findings.

As would be expected, ALSni had higher test scores than both ALSci and ALScbi groups in the majority of cognitive domains, and our sample showed no significant differences in cognitive scores between ALSci and ALScbi. Behavioural changes were assessed with the FrSBe test [24], which comprises three domains: apathy, disinhibition, and executive dysfunction. As anticipated, only the ALScbi group differed from ALSni and ALSci on the total score and the apathy subscale of the test. These neuropsychological results provide context for interpreting the glucose metabolism findings. The explorative non-FWE-corrected comparison of ALSci to ALSni revealed one relatively small hypometabolic cluster in the precentral gyrus despite significant differences between the groups in the majority of assessed cognitive domains. Furthermore, the explorative non-FWE-corrected comparison of ALScbi to ALSci showed a similar distribution of hypo- and hypermetabolic clusters as the FWE-corrected ALScbi-ALSni comparison despite a lack of group differences between ALScbi and ALSci on cognitive scores. Alterations in glucose metabolism may thus be more closely related to the presence of behavioural changes than mild cognitive impairment. However, as behavioural changes cannot be completely separated from cognitive changes, particularly changes in inhibitory control and executive functioning, this somewhat limits the interpretation of our results. Further investigations are required to assess the comparative effects of glucose metabolism on cognitive and behavioural symptoms in ALS.

We observed significant differences in ALScbi when compared to ALSni regarding hypometabolism: the clusters were strongly asymmetric, and largely restricted to the frontal and temporal lobes of the right hemisphere. The notion that impaired behaviour is associated with changes in cerebral glucose metabolism has also been demonstrated by Canosa et al. [16]. Though in contrast to our markedly right hemispheric clusters, their findings showed bihemispheric clusters of negative correlation between glucose metabolism and apathy as determined by a subscale of FrSBe. Whereas their study focussed specifically on correlations of apathy, our study considered behavioural impairment in a more generalised manner. The right lateralisation of hypometabolism in behaviourally impaired ALS in this study is in accord with a tendency for right hemispheric dominance in emotional functioning in the neurobiological literature [34].

Concerning voxel-wise analyses of FDG-PET data respective to individual cognitive domains and their subtests, numerous positive correlations could be observed in the explorative SPM analyses without FWE-correction (height threshold of *p* < 0.001, a minimum cluster size of 100 voxels), whereas corresponding analyses utilising the PLS approach indicated a lack of associations. The diverging nature of these findings suggests an absence of strong correlations between cognitive performance and glucose metabolism at rest. Furthermore, only explorative analyses at a lenient threshold yielded associations with GM volume, which is in agreement with a previous publication [35] that reported small clusters of correlations between GM volume and ECAS scores. Therefore, possible associations between cognition and FDG-PET and GM, if present, seem to be weak.

Several studies reported applying a PVEC [10, 14, 15] and others did not [8, 9, 11, 13, 16]. Here, we directly compared the results of FDG-PET analyses with and without PVEC. The analyses with and without PVEC led to similar results. The clusters identified with both approaches overlapped to a large degree, and the LVs and their significance in the PLS analyses were similar. The influence of PVEs on FDG-PET signals seems to be negligible in ALS samples, and carrying out a PVEC does not seem to affect the results.

We need to consider several limitations of this study. First, a larger sample size would provide higher statistical power to better detect small effects and to give more confidence in interpreting negative findings as absence of true effects. Furthermore, a comprehensive neuropsychological test battery and/or population norms based on larger samples could have yielded more accurate categorisations between the cognitive groups in addition to a more detailed assessment of individual cognitive domains in contrast to the ECAS assessment [10, 13, 15, 36]. Unfortunately, the impact of motor symptoms on test performance cannot be controlled for many cognitive tests and therefore limits the validity of such test findings in ALS patients. The ECAS is able to assess cognition independent of the severity of motor symptoms and has therefore been adapted in the routine assessment of ALS patients in our centre as well as many others. Although the ECAS has become a standard screening instrument for cognitive assessment in ALS owing to its practicability and feasibility placing far less burden on patients with the shorter testing time, our findings suggest that utilising the ECAS for research purposes is less desirable than in clinical routine.

In conclusion, we found that concurrent cognitive and behavioural impairment in ALS was associated with widespread changes in glucose metabolism. In contrast to previous results which suggested notable differences in glucose hypometabolism between ALSni and ALSci groups, in our study, significant differences were only observed in the group comparison between ALSni and ALScbi. In addition, PVEC did not have a conspicuous effect on FDG-PET analyses in an ALS sample, which suggests that the results from publications with and without PVEC are comparable. The individual cognitive domains did not strongly correlate with glucose metabolism or GM volume, but this result should be replicated in larger samples with more comprehensive cognitive assessments.

### Supplementary Information

Below is the link to the electronic supplementary material.

Supplementary file1 (PDF 577 KB)

## Data Availability

The data analysed in the current study can be received from the corresponding author upon reasonable request from qualified investigators.
