# Supplementary material for: Cerebral glucose metabolic correlates of cognitive and behavioural impairments in amyotrophic lateral sclerosis
Source: J Neurol. 2024 Jun 11;271(8):5290–300. doi: 10.1007/s00415-024-12388-z (PMC11319432; doi:10.1007/s00415-024-12388-z)
Supplement: Supplementary file 1 — Supplementary file1 (PDF 577 KB) [file 415_2024_12388_MOESM1_ESM.pdf]

Supplementary material

Supplementary Figure 1. Clusters of relative hypo- and hypermetabolism per group comparison

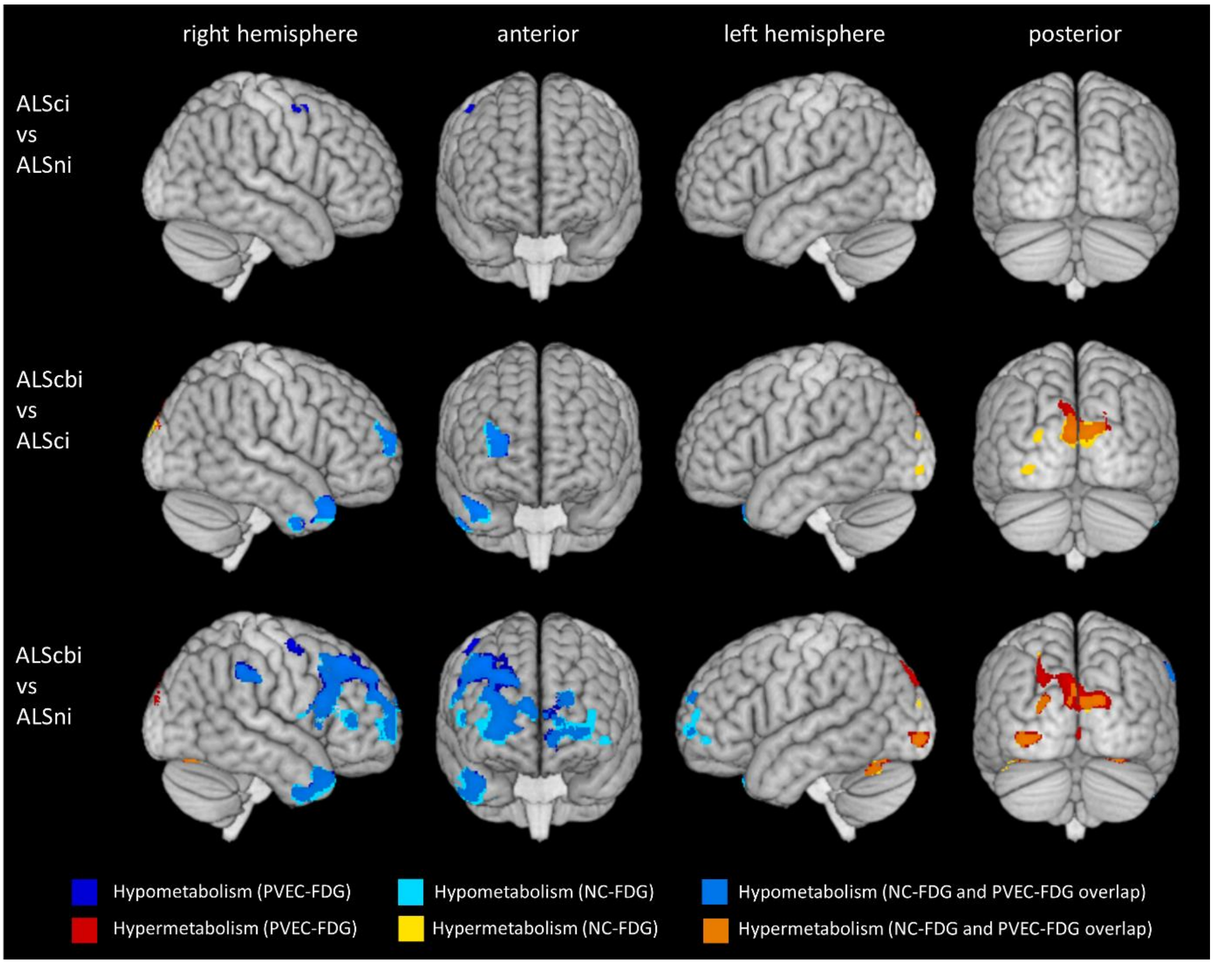

The FDG-PET signal is relative to the average FDG-tracer uptake in GM. Height threshold is $p<0.001$ and minimum cluster size is 100 voxels for all data.

ALSni, not impaired; ALSci, cognitively impaired; ALScbi, cognitively and behaviourally impaired; PVEC-FDG, PVE-corrected FDG-PET data; NC-FDG, non-corrected FDG-PET data.

Supplementary Table 1. Clusters of GM volume associated with cognitive domain scores.

**Labels**	**Side**	**K**	**x**	**y**	**z**	**P (uncorr. at cluster level)**	**P FWE (corr. at cluster level)**
ECAS: Executive functions							
Cerebellum VIII	R	1006	10	-72	-45	**0.023**	0.100
Cerebellum Crus II	R	207	18	-75	-38	0.269	0.709
ECAS: Memory							
Gyrus rectus	R	581	9	36	-28	0.072	0.284
Cuneus	R	317	15	-74	30	0.173	0.550
Precuneus	L	242	-21	-64	16	0.231	0.656
Hippocampus	L	201	-32	-24	-12	0.278	0.724

FWE = family wise error, K = cluster size in voxels, R = right, L = left. Only clusters with more than 200 voxels are presented.
